# Characterization of Nanolayer Intermetallics Formed in Cold Sprayed Al Powder on Mg Substrate

**DOI:** 10.3390/ma12081317

**Published:** 2019-04-23

**Authors:** Sugrib Kumar Shaha, Hamid Jahed

**Affiliations:** Department of Mechanical & Mechatronics Engineering, University of Waterloo, 200 University Ave W, Waterloo, ON N2L 3G1, Canada; hamid.jahed@uwaterloo.ca

**Keywords:** cold spray Coating, magnesium alloy, aluminum powder, bonding mechanism, nanoindentation

## Abstract

Supersonic impact of particles in their solid state with substrate at a low temperature creates a complex bonding mechanism and surface modification in cold spray coating. Here, we report the formation of a layer of 200 to 300 nm intermetallic at the interface of cold spray coated AZ31B-type Mg alloy with AA7075-type Al alloy powder. XRD, SAED, and FFT analysis confirmed the layer possessed BBC crystal structure of Mg_17_Al_12_ intermetallic. The HR-TEM image analysis at the interface identified the BBC crystal structure with interplanar spacing of 0.745 nm for (110) planes, suggesting the Mg_17_Al_12_ phase. The nanoindentation tests showed that the hardness at the interface was ~3 times higher than the substrate. It was also noticed that Young’s modulus at the interface was 117GPa. The combined action of impact energy and carrier gas temperature, along with the multiple passes during coating, caused the formation of intermetallic.

## 1. Introduction

Magnesium (Mg) alloys are the lightest engineering material, and are attractive to automotive, aerospace electronic, and bioimplants industries because of their high specific strength, good damping capacity, and prevalence in nature [1,2,3,4]. The low hardness of Mg may cause material degradation during application due to impact or scratching on the surface of the components [5,6]. The cold spray (CS) technique, which deposits dense and high-quality coatings on the surface of the Mg-alloy parts, has shown promising results in resolving this problem [7]. It has also been shown that the AA7075-type Al alloy (later will be considered as AA7075) coating on AZ31B-type Mg alloy (later will be considered as AZ31B) significantly improves the fatigue properties of Mg alloy substrate [8,9]. Compared to other coating processes, such as anodizing, vapor deposition, electroplating, conversion coating, etc., CS delivers harder and thicker coatings, which can protect Mg alloys in harsh environments [9,10,11]. At the same time, conventional plasma spray process presents challenges in depositing pure aluminum powder due to its combustible nature. In the CS process, small solid particles are accelerated with the help of preheated gas at supersonic velocities, which then impact the substrate. Upon impact with the substrate, the particles undergo severe plastic deformation and form a coating. Here, the critical particle velocity plays a key role in the quality of the coatings. The dense coatings are formed without melting the spray powder or substrate, which, with the help of the kinetic energy of the particles, causes shear interlocking [12,13] or metal jetting [14,15].

The reliable bonding mechanism that occurs during the CS process is still not well understood. The commonly proposed bonding mechanisms of particle/particle or particle/substrate are considered to be mechanical interlocking and adiabatic shear instability, resulting from severe plastic deformation at the interface [16]. Mechanical bonding is characterized as bonding that occurs without any chemical reaction; the coating particles are mechanically trapped by the substrate material to form the interlock. Researchers have used finite element simulation and experimental tools to investigate the bonding mechanism of the CS materials. Assadi et al. [12] and Grujicic et al. [17] showed that the interfacial bond of the particle/particle or particle/substrate interfaces is formed due to the adiabatic shear instability resulting from conversion of the kinetic energy. The interfacial instability may enhance the material mixing at the interface, which is formed through mechanical interlocking between the coating and the substrate [17]. Schmidt et al. [18,19] reported that the bonding in CS is strongly related to the process parameters, such as gas temperature, powder size, particle velocity, etc. There were no shear instabilities observed in particles <5 μm. In contrast, by increasing the particle size (to ~15 μm), the temperature increased, which led to shear instabilities with velocities of 500 m/s or above. Thus, Hassani-Gangaraj et al. [15] recently reported that rather than shear instability, metal jetting is responsible for metallic bonding during CS. In all of the above cases where shear instability was achieved, the peak temperature rose asymptotically with impact velocity up to the melting point. At the same time, it was reported that melting can negatively influence the adhesion under some conditions in the CS process [20]. However, there are a few studies, mainly through numerical simulation, that report local melting during the CS process, in which the possible melting temperature was shown for Al/Cu [21]. The impact-induced melting was identified in the pure Al coatings deposited on the Sn substrate using helium gas with a pressure of 2.5 MPa [18]. Other studies have also identified fusion due to impact during CS of Al–12Si on steel substrate [22]. However, less attention has been paid to the study of the coating/substrate interfacial microstructure of cold sprayed Al alloys on Mg alloys for multiple passes. Zhang et al. [23] investigated the effect of the postspray annealing process on the formation of thick intermetallics at the interface of the AZ91 Mg alloy coated with pure Al using helium as the carrier gas with a temperature of 125 °C and pressure of 620 KPa. They reported that a thick intermetallic layer was formed, which effectively increased wear and corrosion after post processing. Recently, Wang et al. [10] studied the interfacial microstructure of CS coating in a high-speed single pass of a single particle of pure Al on AZ91D Mg alloy using helium as the carrier gas at a temperature of 200 °C and gas pressure of 620 KPa. They identified a mixture of Mg/Al with a ~20 nm thickness at the interfaces. Studies have also showed that the CS of Al alloys on Mg/Al substrate required a higher temperature and higher carrier gas velocity [7,24]; though the interfacial reaction between coating and substrate controlled the bonding strength of the coating materials. However, there was no detailed information about the interfacial microstructure and crystal structure of the CS of AA7075 Al alloy on Mg cast AZ31B processed at a higher carrier gas temperature.

Here, details of the microstructure evolution along the interface of cold sprayed AA7075 coatings on cast AZ31B substrate are reported. A thorough analysis of microstructure and crystallographic relationships was conducted using TEM and XRD. In addition, the mechanical properties of the different phases formed at the interface were measured through nanoindentation.

## 2. Experimental Details

### 2.1. Processing of Coating

High-strength AA7075-alloy [8] spherical powder with an average particle size of 35 μm and cast AZ31B (3.29 wt.% Al, 1.33 wt.% Zn, 0.37 wt.% Mn, and balance Mg) were used as the coating material and substrate, respectively. The CS coating was performed at the Fatigue and Stress Analysis laboratory of the University of Waterloo in Waterloo, Canada, using the Supersonic Spray Technologies (SST) Series P CS system manufactured by Centerline Ltd., (Windsor, Canada). The coating processing parameters are given in Table 1. The carrier gas was nitrogen and the processing temperature was 400 °C with a nozzle stand-off distance of 10 mm. The AA7075 particles were accelerated through the converging–diverging DeLaval nozzle to the supersonic velocities and a low pressure of 200 psi in the CS system. A 200-μm-thick coating was achieved through multiple passes with a step-over of 1.2 mm.

### 2.2. Microstructural Analysis

Thin foils (<100 nm) were prepared using a focused ion beam (FIB, Zeiss NVision40) combining a Schottky field emission SEM. The thin foils were analyzed using transmission electron microscopy (TEM), performed in a JEOL-2010F equipped with energy-dispersive X-ray spectroscopy (EDX) and an operating voltage of 200 keV. HR-TEM images, high-angle annular dark-field (DF) scanning transmission electron microscopy (HAADF-STEM) imaging, selected area electron diffraction (SAED), and fast Fourier transformation (FFT) patterns were analyzed using CrysTbox [25].

The phase identification (PID) at the polished cross-section was studied using a Bruker D8-Discover equipped with a VÅNTEC-500 area detector and Cu-Kα radiation at a voltage of 40 kV and current of 40 mA. The measurement was performed in three locations at the polished cross-section using a 300-µm collimator. First, the measurement was performed on the substrate. The collimator was then moved to the interface and finally to the coating, to complete the measurement. The three sets of data were evaluated using Bruker’s Diffrac.Eva software. Details of the XRD analysis are described in [26].

### 2.3. Nanoindentation Testing

Nanoindentation was performed using a Hysitron Triboindenter TI-900 equipped with a scanning probe microscope and an indenter size of 100 nm. The test was performed at a constant loading rate of 500 μN/s up to a maximum load of 1000 μN. At least 5 indents were made on the coating, interface, and substrate; the average measured values were compared with bulk data reported in the literature. 

## 3. Results

### 3.1. Microstructure

**Figure 1:**
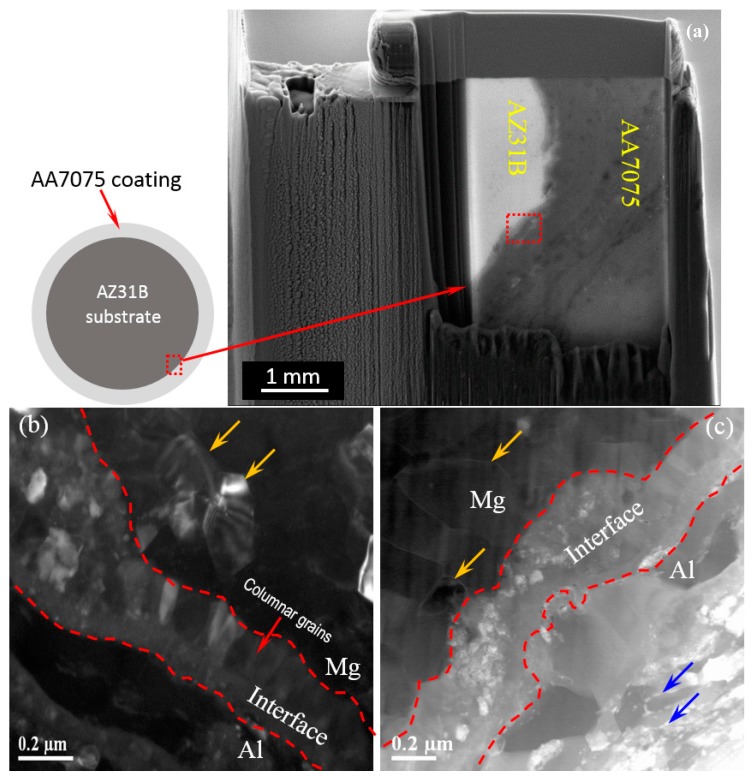
A schematic cross-sectional view of the coated sample along with (**a**) the TEM membrane, prepared by focus ion beam (FIB) milling; (**b**) the dark-field DF image; and (**c**) the high-angle annular dark-field (DF) scanning transmission electron microscopy (HAADF-STEM) image of the Al7075 cold spray coating on the AZ31B substrate. The dotted line shows the boundary of the interface between the substrate and the coating. The area enclosed by the red line in a is presented in Figure 2.

Figure 1a shows the SEM image of a sample prepared for transmission electron microscopy (TEM) analysis using focus ion beam (FIB) milling. Three distinct zones in the cross-sectional FIB membrane are shown, and can be seen more clearly in the higher magnification high-angle annular dark-field (DF) scanning transmission electron microscopy (HAADF-STEM) images near the interface (Figure 1b,c). The red dashed lines in Figure 1b,c distinguish the three zones: the Mg-substrate, the interface, and the AA7075-coating. A continuous layer of 200–300 nm was identified between the coating and the substrate. The grain size obtained near the interface was ~200 nm after, as indicated by the yellow arrows in Figure 1b,c, while the grain size of the as-cast substrate was ~278 µm [27]. This is an indication of grain refinement and the formation of nano-structures due to CS. The elongated ladder-like grains are seen in AA7075-coating with MgZn_2_ precipitates along the grain boundaries, as indicated by the blue arrows in Figure 1c. Also, a columnar grain size of ~100 nm is observed in the interfacial region (Figure 1b), which can be an indication of the direction of heat flow during rapid solidification [28]. A similar type of columnar grains was reported for the forming of coextruded Al–Mg sheet at the interface under different load conditions [29]. The columnar grains may be formed by the high temperature gradient and high solidification rates. The high solidification rate is achieved due to strain-induced melting followed by rapid quenching [30].

**Figure 2:**
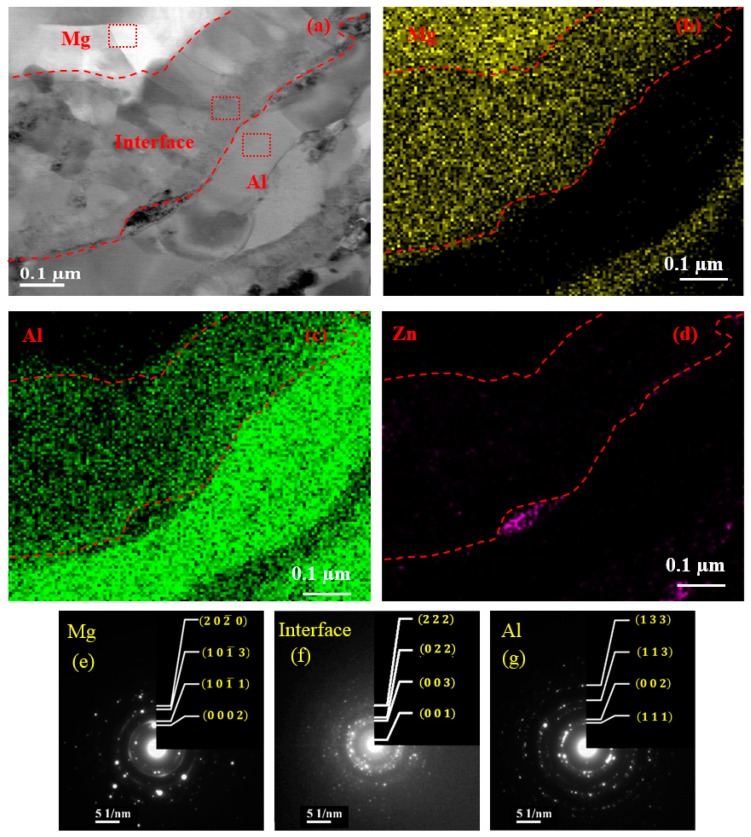
Typical bright field-transmission electron microscope (BF-TEM) microstructure (**a**) near the interface along with the EDX image mapping (**b**–**d**) and the SAED pattern (**e**–**g**) of the corresponding zones, separated by the red dashed lines.

Figure 2a shows the bright field BF-TEM image with EDX-image mapping and the corresponding selected area electron diffraction (SAED) pattern near the interface (the area enclosed by the red box in Figure 1a). EDX image mapping of the interfacial zone exhibits the distribution of both Al and Mg elements, while Zn is only visible at grain boundary as MgZn_2_ precipitates. Also, the average concentration of the Mg and Al obtained using EDX in the interfacial area of 1600 nm^2^ is ~56.37 ± 1.2 and 43.63 ± 1.2 wt.%, respectively. It should be mentioned that the average concentration of the Zn is ~0.10%. As marked by the red boxes in different zones in Figure 2a, the SAED patterns show polycrystalline structure matching with Mg, Mg_17_Al_12_, and Al phases, which correspond to the substrate (Figure 2e), interface (Figure 2f), and coating (Figure 2g), respectively.

**Figure 3:**
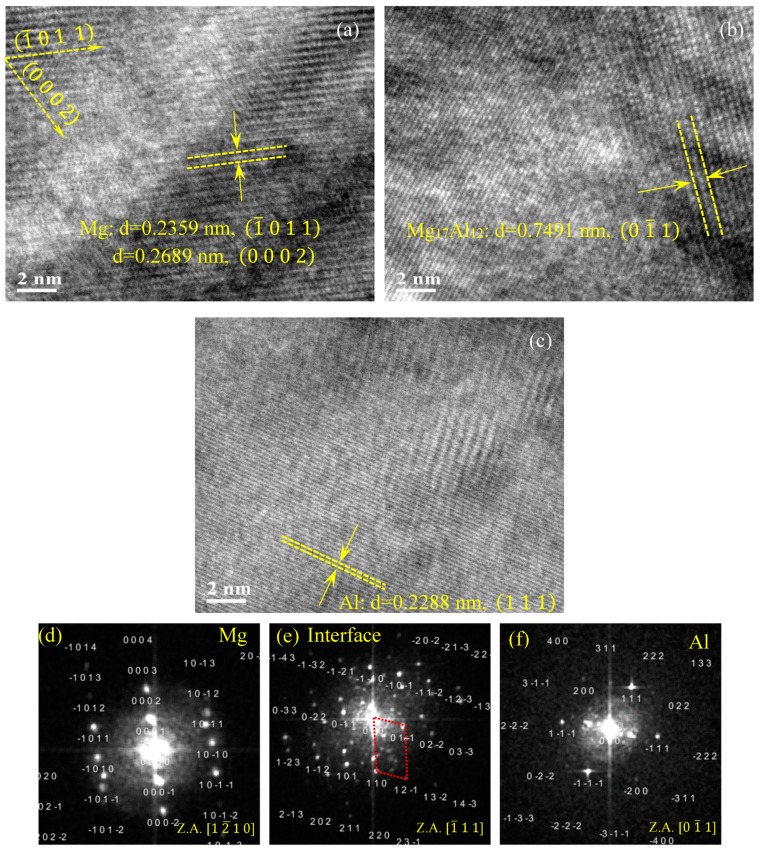
Typical HRTEM images of different zones and the fast Fourier transform (FFT) diffraction patterns of cold sprayed AA7075 on the AZ31B substrate: Mg substrate (**a**,**d**), interface (**b**,**e**), and AA7075 coating (**c**,**f**), separated by the red dashed line in Figure 2.

To further analyze the details of the crystal structure near the coating/substrate interface of the CS process, HR-TEM images showing lattice fringes and corresponding FFT patterns were examined. Figure 3 illustrates typical HR-TEM images acquired from the coating, interface, and substrate, which are specified by red rectangles on the BF image in Figure 2a. A complete order structure with different interplanar spacing is observed in different zones. As there was no distortion of atoms in the AA7075-coating and AZ31B-substrate during the CS, full lattice matching between the Al and Mg was identified in the coating and Mg substrate, respectively. Following the analysis of the SAED (Figure 2e–g) and the corresponding FFT images (Figure 3d–f), it is observed that the zonal axis of the substrate is [1$\bar{2}$10], and the angle between the (0002) and ($\bar{1}011$) planes is 61.92° with interplanar spacing of 0.2689 nm and 0.2359 nm, respectively, which indicates an HCP structure of Mg alloys. In contrast, the FCC crystal structure was identified in Al-base coating where the d-spacing of the (111) plane was 0.2288 nm at the zonal axis of [01$\bar{1}$]. Similar interplanar spacing for the HCP and FCC crystal structure for Mg and Al was reported in [31].

**Figure 4:**
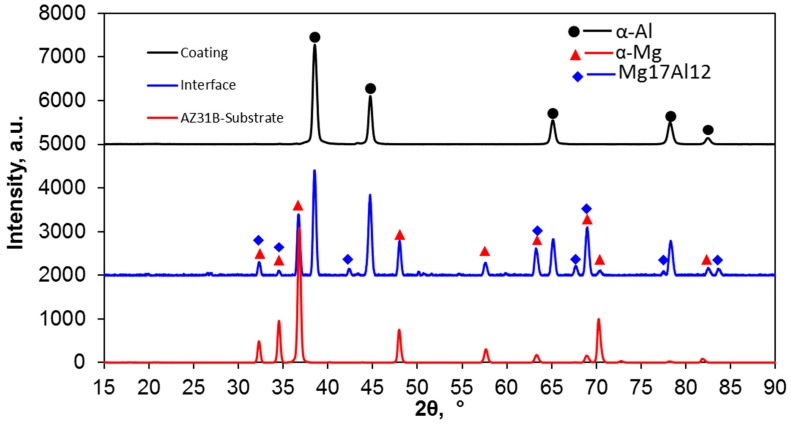
The XRD patterns of the AZ31B Mg alloy coated with AA7075 alloy in coating interface.

Accordingly, the identified BCC crystal structure at the interface and measured interplanar distance of 0.7491 nm is close to the lattice spacing of ($0\bar{1}1$) planes of the intermetallic phase Mg_17_Al_12_ (Figure 4e), which is also in agreement with the literature and SAED results identifying the intermetallic Mg_17_Al_12_ phase (Figure 2f). Hai et al. [32] found that the d-spacing of the crystal plane (110) for Mg_17_Al_12_ was 0.735 nm. Wang et al. [33] also reported that the interplanar spacing of the (110) plane for the Mg_17_Al_12_ phase was 0.7456 nm, for the β-phase formed in AZ91D Mg alloy.

Figure 4 shows the XRD patterns obtained from the substrate, interface, and coatings. It can be seen that α-Al and α-Mg phases were identified in the coating and substrate, respectively. However, the Mg_17_Al_12_ phase with α-Al and δ-Mg phases (same as α-Mg) was recognized at the interface. It should be noted, however, that while the collimator size was 300 µm, multiple measurements by successive advancement of the collimator from the substrate to the interface and coating were made. To analyze the measurements at the interface, the intensity of α-Mg and α-Al were incorporated at the interfacial XRD pattern. It should be mentioned that the volume fraction and size of the intermetallics are lower than the XRD detectable range. So, intermetallics containing Zn, Mn, and Cu were not identified. From the XRD results it can also be concluded that the Mg_17_Al_12_ phase was formed at the interface. Nie et al. [34] studied the interfacial structure of Al/Mg/Al laminates. They reported a similar type of Mg_17_Al_12_ phase at the interface with cubic crystal structure. Zhang et al. [23] also investigated the interfacial layer formed during annealing after CS processing. A layer of the Mg_17_Al_12_ phase, similar to that reported here, was identified using XRD.

### 3.2. Properties Obtained in Nanoindentation

**Figure 5:**
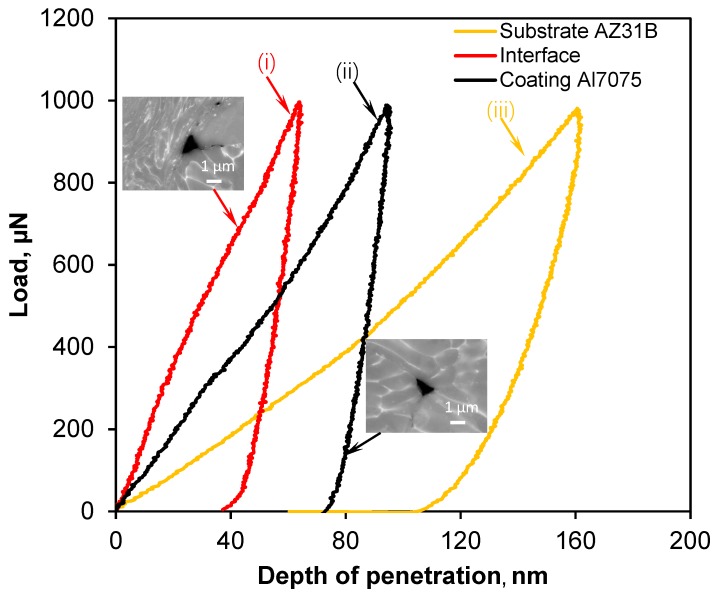
Typical loading–unloading curves obtained during nanoindentation at a load of 1 mN for (i) AA7075 CS coating, (ii) near the interface of AA7075 coating, and (iii) AZ31B substrate, with the corresponding SEM images of indent on the coating and near the interface.

Mechanical properties of the substrate, interface, and coating were investigated using a nanoindenter and compared with the available data in the literature. The load–depth (P–h) curves during nanoindentation on the different zones—substrate near interface, interface, and coating near interface—and their corresponding indent impressions (inserted) are shown in Figure 5. The nanohardness was highest near the interface (3.36 ± 0.82 GPa). Conversely, the nanohardness values of the coating and substrate were 2.13 ± 0.42 and 1.22 ± 0.21 GPa, respectively. Subsequently, the obtained modulus of the substrate, interface, and coating were 37.5 ± 5.21, 117.77 ± 3.51, and 72.46 ± 2.11 GPa, respectively. However, the deformation of the substrate was more severe, as indicated by a higher indent depth (ID) (136.21 ± 5.56 nm) compared to the average ID (68.1 ± 5.19 nm) at the interface, due to a lower modulus and hardness of the substrate. A similar nanohardness value for the Mg_17_Al_12_ intermetallic was reported by multiple researchers [23,35]. Zhang et al. [23] studied the nanomechanics of Mg–Al intermetallics formed during the post processing of pure Al on the AZ91 Mg alloy. Their investigation showed that nanohardness of 4.40 ± 0.3 GPa and 1.24 ± 0.1 GPa were achieved for the Mg_17_Al_12_ intermetallic and Mg substrate, respectively. Mathur et al. [35] also studied the deformation behavior of the γ-Mg_17_Al_12_ phase and measured a room temperature nanohardness value of 3.5 GPa. Higher elastic modulus and hardness suggests stronger bonding between the intermetallic and coating/substrate, and, when the electronegativity difference of two elements is higher than the 1.7 eV, they can be formed by ionic bonding. The electronegativity difference between Mg and Al is 0.3. So, there was no possibility of forming ionic bonding between Al and Mg. Therefore, the bond between the intermetallic Mg_17_Al_12_ and coating/substrate supposed to be a mixture of covalent bonds and metallic, resulting higher modulus of elasticity of the intermetallic.

## 4. Discussion

There are two main features observed in the TEM and XRD analysis of the interface microstructures presented here. These features are (1) the existence of a 200–300-nm-thick Mg_17_Al_12_ intermetallic interlayer and that (2) the interlayer possesses a columnar grain. The formation of this interlayer depends on the deformation behavior of the coating and substrate, different loading conditions, the diffusion time, and the temperature. Wang et al. [10] observed different deformation behavior between the pure Al coating and AZ91D-substrate at a high strain rate, which is responsible for their crystal structure and stacking fault energy (SFE). Al possesses a FCC crystal structure having twelve slip systems with a high SFE of 200 mJ/mm^2^ [36]. In contrast, Mg has an HCP crystal structure with three slip systems, and a lower SFE of 78 mJ/mm^2^ [37]. In Al alloys, deformation occurred in slip by forming dense dislocation walls (DDWs) and dislocation arrays (DAs), which causes the dynamic recrystallization/recovery, leading to grain refinement. However, the presence of fine precipitates (Figure 1c) at the grain boundaries hindered the grain refinement by stopping the movement of grain boundaries, resulting in dynamic recovery. In contrast, twining is activated in Mg alloys for homogeneous deformation, which leads to dynamic recrystallization instead of recovery [38]. The recrystallization of metals induced by the impact energy caused the migration of high-angle grain boundaries and rearrangement of dislocations within the parent crystals. However, in the CS process, the crystallization of the amorphous structure or melts necessitated atomic rearrangement, which required higher temperatures and longer time periods to complete the subsequent impact. Many researchers [18,19,39] have modeled the CS process using FEA to predict the temperature distribution at the interface of the coating/substrate. They reported that the local temperature at the interface is much higher than the coating temperature. The temperature increase at the interface is due to the energy release caused by severe plastic deformation of the particles and substrate at the impact location during CS. The increase in temperature can lead to atomic diffusion across the interface [18,19,39]. Xiong et al. [30] and Wang et al. [10] calculated an atomic diffusion distance of ~0.1 nm during CS. In their studies [10,30], helium was used as the carrier gas, where the intermixing zone was reported to be 15–20 nm. They argued that an intermixing amorphous layer at the interface of the coating/substrate metals could not be formed due to the fast quenching of strain rate-induced melts and solid-state amorphization of incident metals [10,30]. As the investigated interface was single-particle, the time and temperature were insufficient to complete the diffusion in the CS process. In contrast to these studies [10,30], the CS in the present study was performed at a higher temperature with multiple passes, which achieved a longer time and higher temperature to complete the chemical reaction by diffusion resulting in the formation of the Mg_17_Al_12_ intermetallic. Zhang et al. [23] and others [40] reported that due to the diffusion of Al/Mg, an intermetallic layer having Mg_17_Al_12_ was formed during annealing at the interface of the cold sprayed pure Al on the AZ91. Further, carrier gas also influences the formation of the interfacial layer during CS. The AA7075 coating deposited using nitrogen as the carrier gas imposed higher compressive stress than that sprayed with helium due to the enhancement provided by helium, which better accommodated the shear instability or metal jetting [41]. Such nonequilibrium phenomena control the impact fusion during CS, increasing the bonding efficiency [41]. When the interface experienced local melting, it solidified very quickly, resulting in the formation of an amorphous/crystalline structure [30]. However, during subsequent passes, the amorphous region and crystalline phase can transform and grow further due to higher temperatures and atomic diffusion (Figure 1b). According to the Mg–Al equilibrium binary phase diagram, the Mg_17_Al_12_ eutectic phase formed at a temperature of 437 °C. At the same time, AA7075 alloy started to melt at a temperature of 477 °C. The induced high strain energy due to impact and local plastic deformation, along with the carrier gas temperature (400 °C can produce a high enough temperature for local melting, which leads to the formation of the Mg_17_Al_12_ eutectic phase under nonequilibrium conditions.

It is well established that during the CS process, severe plastic deformation is induced, which causes share instability in the coating, and metal jetting in the substrate results in a buildup of residual stress. At the same time, during CS the substrate remains at room temperature, which causes a significant temperature gradient at the interface. It has also been reported that a higher thermal gradient changes the crystal growth from epitaxial to the columnar. When the thermal gradient is above 10^6^ K/m, columnar grains are formed in most of the metals [42]. In the CS process, very high strain rates (~10^9^ s^−1^) and fast cooling rates (~10^10^ K/s) can be achieved at the interface [43]. The high thermal gradient of the substrate may result in columnar grains forming in the substrate rather than in the coating. In addition, the melting point of materials decreases with an increase in strain rate [30], and hence makes melting possible at lower temperatures. Results of another study [16] showed that when only a single particle is deposited, the coating/substrate interfacial stress (IS) increases quickly, and decreases to zero after particle deposition. Consequently, when multiple particles are deposited onto the substrate, the subsequent particles impacted on the former deposited particles results in an incremental increase in IS. The IS of coating/substrate is sustained at a comparatively high level [16]. This high level of IS results in an increase in the interfacial temperature, which may lead to melting and the formation of columnar grains. At the same time, the Kirkendall effect also influences the growth of the interface [44]. The movement of the interface between two phases may happen due to the difference in diffusion rates of the phases’ atoms. Thus, the Al atoms (FCC) diffuse into the Mg (HCP) crystal lattice, which results in the gradual formation of Mg_17_Al_12_, by changing the HCP structure to the BCC structure and the crystal growth from epitaxial to the columnar.

## 5. Conclusions

From the above results and discussion, it is seen that the interface contains Mg_17_Al_12_, which is the main objective to characterize interfacial layer. The localized melting at the coating/substrate interface during CS of AA7075 on cast AZ31B was observed and investigated in this paper. The combined action of carrier gas temperature, heat release due to local severe plastic deformation, high strain rates of impacted particles, reactivity of substrate material, and multiple passes of coating led to the formation of nanograins and a nanolayer of Mg_17_Al_12_. It was shown that particles in this 200–300 nm region have a BCC crystal structure and exhibit columnar grains. Furthermore, the lattice d-spacing of the BCC structured particles within the interface obtained from SAED, and the corresponding FFT was shown to be close to the reported Mg_17_Al_12_ spacing. Also, the high modulus of 117.77 GPa of these particles at the interface as measured by nanoindentation supported the formation of nanograins and intermetallics at the interface.

## Figures and Tables

**Table 1:** Cold spray deposition parameters used in this study.

Carrier Gas	Nitrogen
Gas pressure	1.4 MPa (200 psi)
Gas temperature	400 °C
Nozzle travel speed	5 mm/s
Stand-off distance	10 mm
Step over	1.2 mm
Nozzle type	Converging-diverging DeLaval nozzle UltiLife (Steel)
Nozzle length	120 mm
Nozzle orifice diameter	2 mm
Nozzle exit diameter	6.3 mm
Powder feeder rate	5 rpm (8 gr/min)
Nozzle type	UltiLife TM
